# The effects of crizotinib in a transgenic *Drosophila* model expressing the human TPM4-ALK fusion gene or TPM4

**DOI:** 10.1242/bio.044362

**Published:** 2019-07-15

**Authors:** Yoo Jin Kim, A-Ri Cho, Hee Jung Sul, Bohyun Kim, A-Young Kim, Hyeong Su Kim, Jong Bok Seo, Youngho Koh, Dae Young Zang

**Affiliations:** 1 Hallym Translational Research Institute, Hallym University Sacred Heart Hospital, Anyang, 14066, Republic of Korea; 2 Department of Molecular Medicine, Graduate School of Hallym University, Chuncheon, Gangwon-do, 24252, Republic of Korea; 3 Department of Biomedical Gerontology, Ilsong Institute of Life Sciences, Hallym University, Anyang, Gyeonggi-do, 14066, Republic of Korea; 4 Division of Hematology-Oncology, Department of Internal Medicine, Hallym University Medical Center, Hallym University College of Medicine, Anyang-si, Gyeonggi-do 14068, Republic of Korea; 5 Korea Basic Research Institute Seoul Center, Seoul, 02855, Republic of Korea

**Keywords:** Crizotinib, *Drosophila*, Cancer, Fusion gene (TPM4-ALK)

## Abstract

Anaplastic lymphoma kinase (ALK) fusion events lead to constitutive activation of the ALK kinase domain, thereby functioning as oncogenic drivers. These fusion proteins have been identified in numerous cancers. Crizotinib, a small molecule inhibitor of c-Met and ALK, is a Food and Drug Administration-approved drug with reported efficacy in the treatment of cancer. Tropomyosins (TPMs) are a family of actin filament-binding proteins. Altered TPM expression has been found in a variety of human tumors. Inhibitors of cancer-associated TPMs and actin-targeting compounds have been developed, but anti-actin agents have cardiac and respiratory muscle toxicities. In this study, we investigated the sensitivities of human TPM4 (hTPM4), human ALK (hALK), and their fusion gene (hTPM4-hALK) to crizotinib by measuring the lifespan of transgenic *Drosophila*. Flies overexpressing hTPM4-hALK, hTPM4 and hALK showed decreased lifespans compared with controls. Although crizotinib is an inhibitor of ALK, treatment with crizotinib significantly extended the lifespans of *Drosophila* expressing hTPM4 and hTPM4-hALK but had no effect on hALK-expressing flies. Autophosphorylation of Tyr^1278^ is necessary for full activation of the ALK domain. We confirmed that hTPM4-hALK was phosphorylated at Tyr^1278^ in a ligand-independent manner, and hTPM4-hALK-expressing flies treated with crizotinib showed a decreased level of Tyr^1278^ phosphorylation compared with untreated hTPM4-hALK-expressing flies, with a greater decrease induced by 1 µM compared with 200 nM crizotinib. Taken together, the results suggest that crizotinib is effective for treating ALK-driven cancer and might be a new therapeutic drug, without cardiac or respiratory muscle toxic effects, for TPM4-expressing cancers.

## Abstract (precis)

**Summary:** In this study, we find that crizotinib extends the lifespan of *Drosophila* overexpressing hTPM4-ALK and hTPM4, which are linked with a variety of human tumors.

## INTRODUCTION

Gastric cancer is one of the most common cancers and is the second leading cause of cancer-related death worldwide. A variety of genetic alterations in TP53, PIK3CA and KRAS have been identified, and many molecular targeted drugs have been evaluated for treatment of gastric cancer (Yang et al., 2014). Here, we assessed the effects of crizotinib using a *Drosophila* model. Because of its relatively short lifespan (approximately 2 months) and conservation of human disease-related genes, *Drosophila melanogaster* has been widely used as an *in vivo* tool for validation or discovery of effective cancer drugs (Sun et al., 2013; Yadav et al., 2016). Recent advances in molecular genomics have identified various oncogenic gene alteration, one of the most important types being the fusion of two genes (Parker and Zhang, 2013). Among various fusion genes involved in cancers, anaplastic lymphoma kinase (ALK) has been found fused to various genes in diverse cancers (Hallberg and Palmer, 2013). ALK is a receptor tyrosine kinase that was first identified as a fusion gene with nucleophosmin 1 (NPM1-ALK), via a 2;5 chromosomal translocation in anaplastic large-cell lymphoma (Morris et al., 1995). ALK functions as an oncogenic driver upon its gene amplification, introduction of point mutations and formation of fusion genes. A variety of ALK fusion genes resulting in constitutive activation of ALK, which is normally regulated by its extracellular ligand-binding domain in the full-length molecule, have been identified in human cancers (Grande et al., 2011; Hallberg and Palmer, 2013). Tropomyosins (TPMs) constitute a family of actin filament-binding proteins that were first identified in studies of skeletal muscle contraction. Human cells contain four tropomyosins: TPM1, TPM2, TPM3 and TPM4. Altered tropomyosin expression has been found in a variety of human cancers, including breast, colon, lung, prostate, ovary and gastric cancers (Stehn et al., 2006; Choi et al., 2012). TPM3-ALK and TPM4-ALK, which are fusion proteins consisting of the N-terminus of TPM and the C-terminal kinase domain of ALK, have been reported in patients with inflammatory myofibroblastic tumors, with TPM3-ALK being the most frequently observed (Lawrence et al., 2000). Crizotinib is a small-molecule selective inhibitor of c-Met and ALK tyrosine kinases. It was approved by the Food and Drug Administration for the treatment of ALK-positive non-small-cell lung cancer and inflammatory myofibroblastic tumors (Butrynski et al., 2010; O'Bryant et al., 2013). In a previous study, we identified the TPM4-ALK fusion gene in gastric cancer tissues of Korean patients (unpublished). In the present study, we evaluated the sensitivity of the TPM4-ALK fusion gene to crizotinib, using transgenic *Drosophila* expressing the human TPM4 (hTPM4)-human ALK (hALK) fusion gene. We also assessed the sensitivities of the full-length hALK and hTPM4 genes to crizotinib in transgenic *Drosophila*. The results suggested that the hTPM4-hALK fusion gene is constitutively activated, resulting in early death of *Drosophila*. We also found that the activity of hTPM4-hALK was inhibited by treatment with crizotinib.

## RESULTS

### Molecular characteristics of hTPM4, hALK and hTPM4-hALK and their and ectopic expression in *Drosophila*

**Fig. 1.**
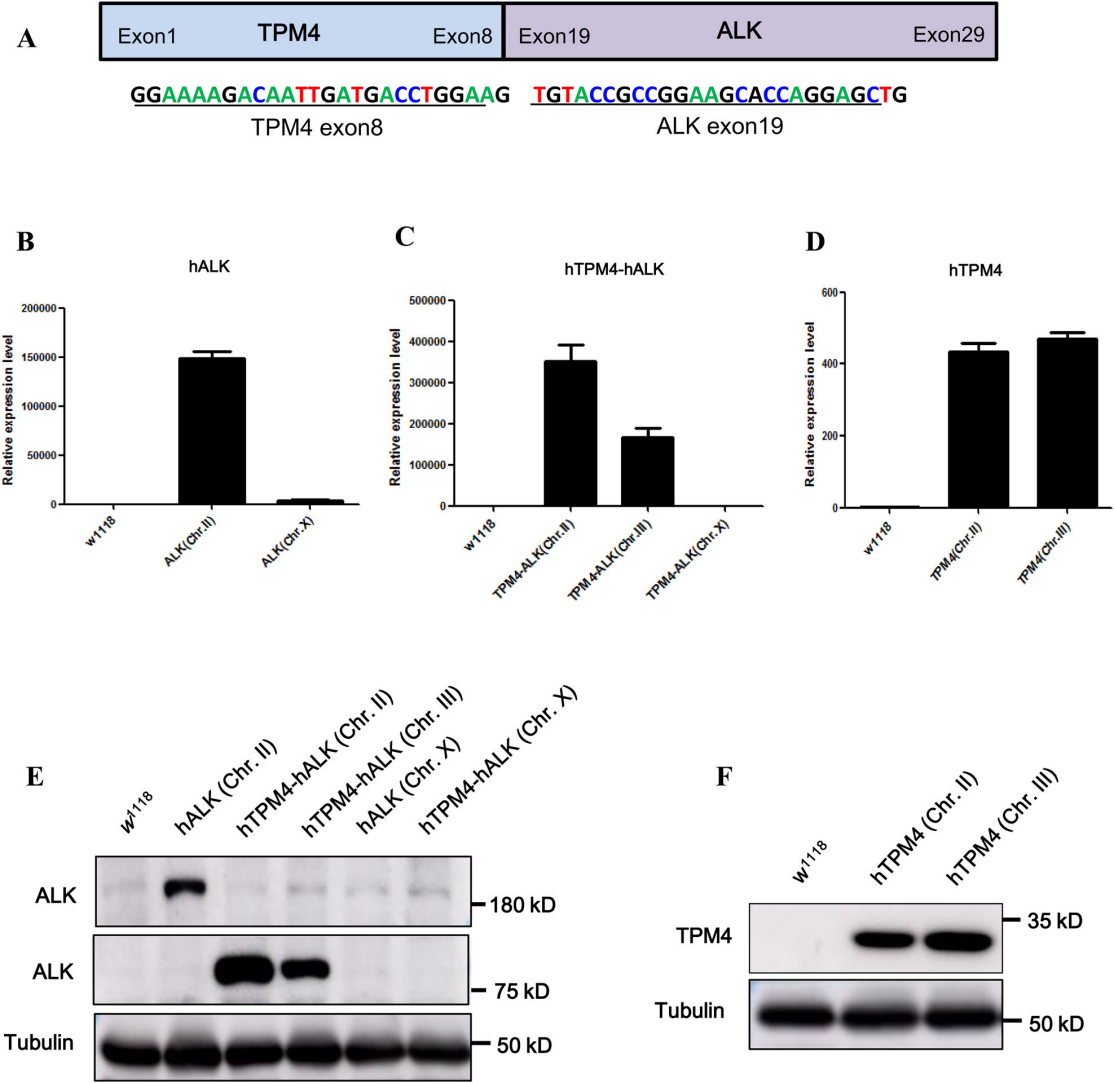
**Generation of transgenic flies.** (A) Schematic representation of the hTPM4-hALK fusion protein. The hTPM4-hALK fusion gene involves fusion of hTPM4 exon 8 to hALK exon 19, comprising the N-terminal domain of hTPM4 and C-terminal kinase domain of hALK. The (B–D) mRNA and (E,F) protein expression of hALK, hTPM4-hALK and hTPM4 in male flies overexpressing each of these genes was measured by qRT-PCR and western blot analysis, respectively.

In previous studies we characterized fusion proteins in the tumor tissues of gastric cancer patients. Among these selected fusion proteins, we found that the TPM4-ALK fusion protein is preferentially expressed in gastric cancer tissues compared with normal tissues. We subsequently identified the amino acid sequence of the TPM4-ALK fusion protein (unpublished), which showed that exon 8 of TPM4 is fused to exon 19 of ALK (Fig. 1A). To investigate whether the lifespan of *Drosophila* is sensitive to crizotinib as mediated by hTPM4-hALK, we generated transgenic *Drosophila* expressing hTPM4, hALK and hTPM4-hALK. Expression of these genes in *Drosophila* was confirmed by real-time reverse-transcription polymerase chain reaction (qRT-PCR) and western blot analysis. As shown in Fig. 1, the mRNA expression of hALK, hTPM4-hALK and hTPM4 was observed in two or more independent transgenic lines for each gene construct. Protein expression was confirmed by western blot analysis using antibodies specific to ALK and TPM4 (Fig. 1E,F).

### Expression of hALK, hTPM4 and hTPM4-hALK decreased the lifespan of transgenic *Drosophila*

**Fig. 2.**
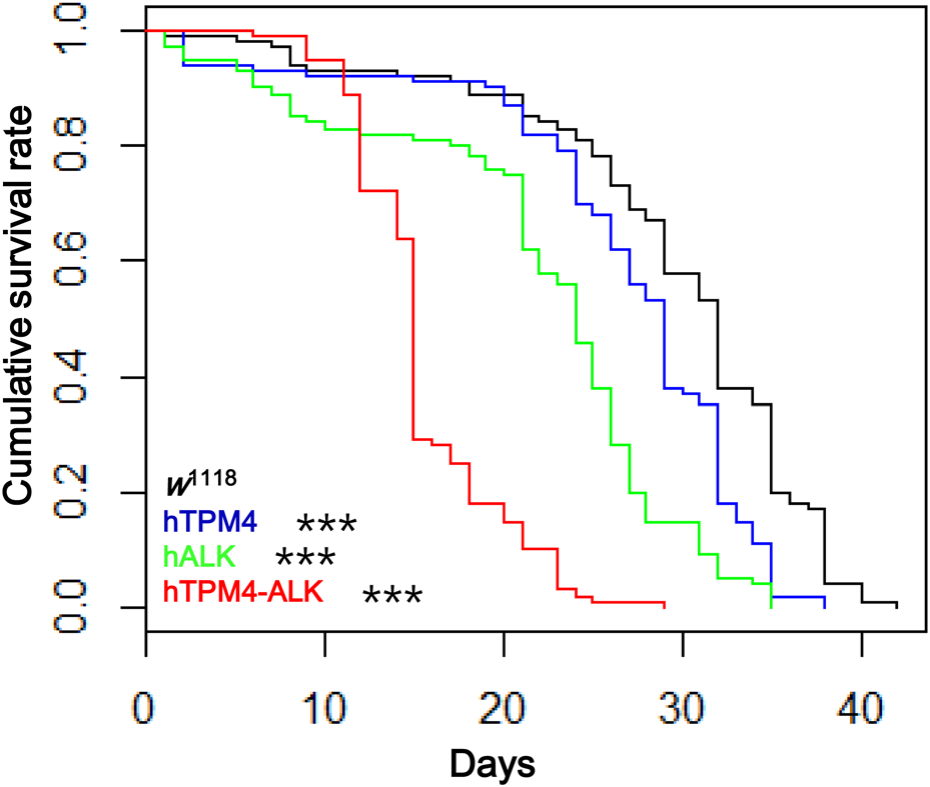
**Lifespan analysis of transgenic flies grown on normal food.** The hTPM4-hALK fusion transgenic male flies showed a significantly shorter lifespan compared with control *w*^1118^ flies when grown on standard food. The hALK- and hTPM4-overexpressing flies also showed decreased lifespans. Survival was more decreased in male than female flies. Log-rank analysis showed significant differences between the transgenic flies and *w*^1118^ flies. ***P<0.001.

We evaluated whether overexpression of hTPM4, hALK or hTPM4-hALK had an effect on lifespan in *Drosophila*. Survival analysis using the Kaplan–Meier method showed that the lifespan of flies expressing hTPM4-hALK was shorter than that of *w*^1118^ control flies (hazard ratio [HR]: 17.11; *P*<0.001). The lifespan of flies expressing hALK (HR: 3.70; *P*<0.001) or hTPM4 (HR: 1.83; *P*<0.001) was significantly decreased compared with that of control flies (Fig. 2, Table 1). The mean lifespan of the flies expressing hTPM4-hALK was 15.24±0.85 days compared with 37.56±0.87 days for control flies. The maximum lifespan of the hTPM4-hALK transgenic flies was 24 days compared with 45 days for control flies. The mean lifespan of the hTPM4- and hALK-expressing flies was 27.36±1.20 and 25.98±0.82 days, respectively (Table 2).

**Table 1.**
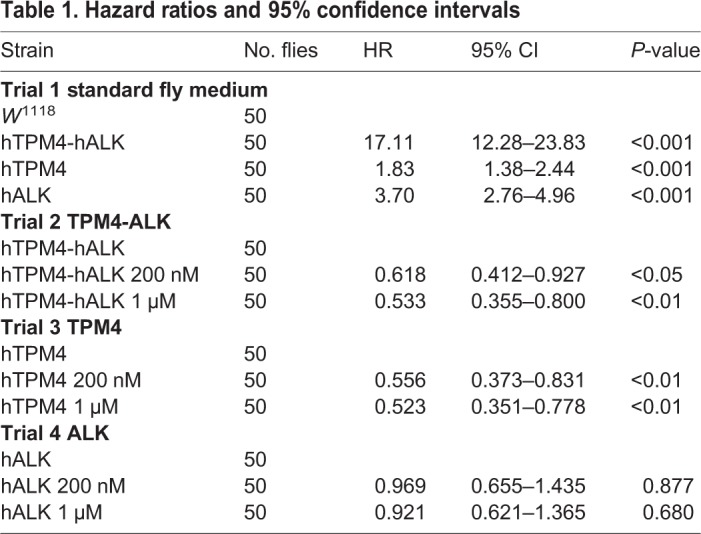
**Hazard ratios and 95% confidence intervals**

**Table 2.**
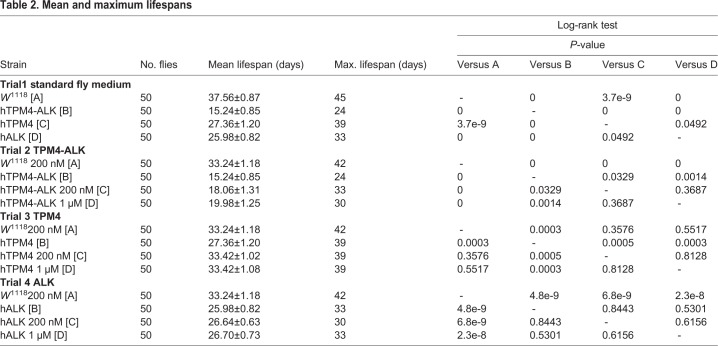
**Mean and maximum lifespans**

### Crizotinib increased the lifespan of transgenic *Drosophila* expressing hTPM4-hALK

**Fig. 3.**
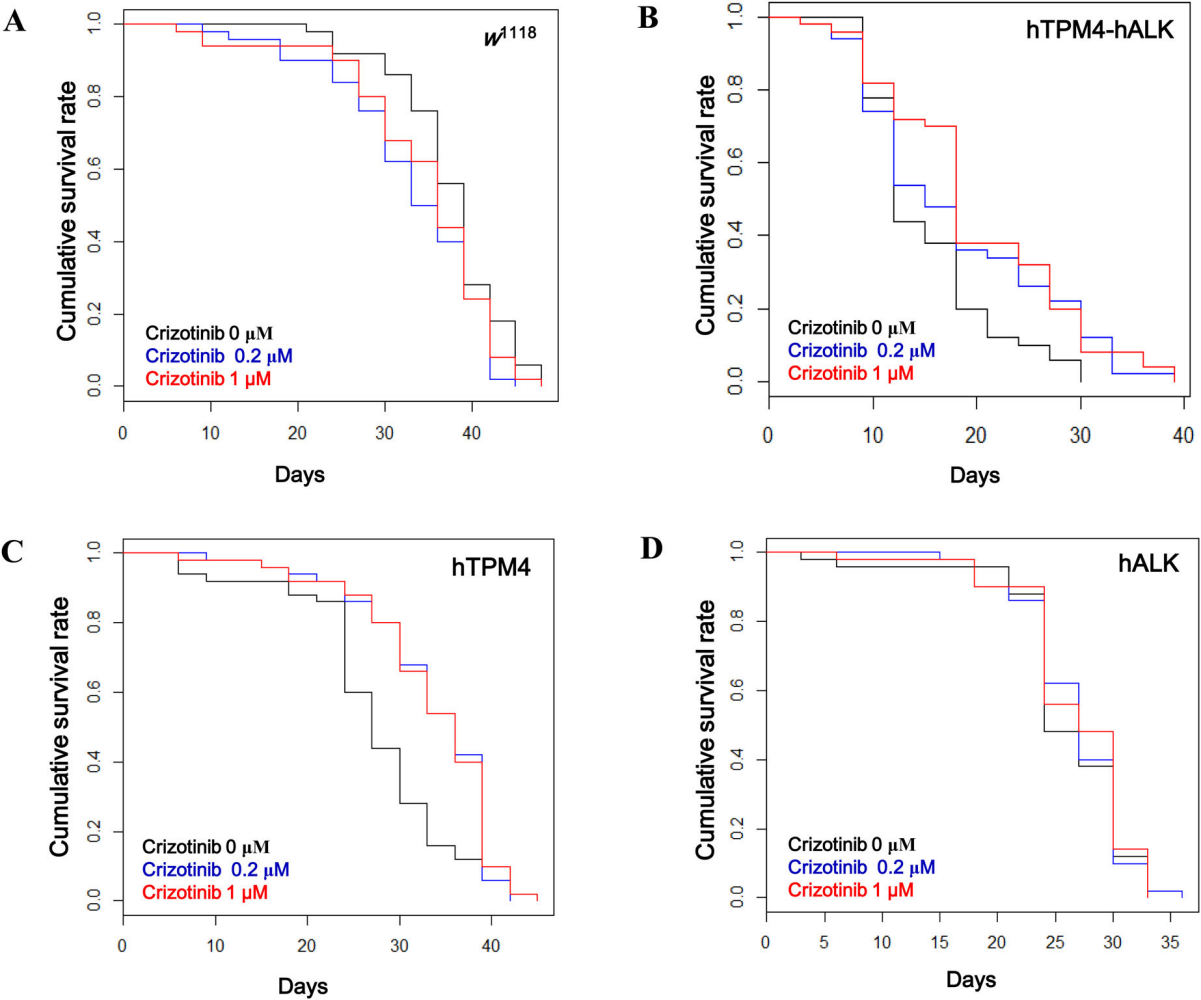
**The effect of crizotinib on the survival of control and transgenic flies.** (A) The lifespan of *w*^1118^ male flies was similar between the low (200 nM) and high (1 μM) doses of crizotinib. (B) Both 200 nM and 1 μM crizotinib increased the lifespan of male flies expressing hTPM4-hALK. (C) Both 200 nM and 1 μM crizotinib increased the lifespan of male flies expressing hTPM4. (D) Crizotinib had no effect on the lifespan of male flies expressing hALK.

We first evaluated the toxicity induced by crizotinib in flies by treating *w*^1118^ flies with 200 nM or 1 µM crizotinib and measuring the lifespan of the flies (Fig. 3A). Crizotinib did not have any effect on the lifespan of the flies, indicating that crizotinib is not toxic at these concentrations. Next, to assess the effect of crizotinib on survival in the presence of hTPM4-hALK overexpression, we monitored the lifespans of transgenic flies treated with 200 nM or 1 µM crizotinib*.* Crizotinib increased the survival of flies expressing hTPM4-hALK at both 200 nM (HR: 0.618; *P*<0.05) and 1 μM (HR: 0.533; *P*<0.01) (Fig. 3B, Table 1). The mean lifespan of flies expressing hTPM4-hALK treated with 200 nM or 1 µM crizotinib was 18.06±1.31 or 19.98±1.25 days, respectively, compared with 15.24±0.85 days for the untreated hTPM4-hALK-expressing flies. The maximum lifespan of flies expressing hTPM4-hALK treated with 200 nM or 1 µM crizotinib was 33 or 30 days, respectively, compared with 24 days for the untreated hTPM4-hALK-expressing flies (Table 2). Taken together, the results suggested that crizotinib has an anti-cancer effect in transgenic *Drosophila* expressing hTPM4-hALK, as evidenced by an extension of lifespan.

### Crizotinib increased the lifespan of transgenic *Drosophila* expressing hTPM4

We also evaluated the effect of crizotinib on the lifespan of transgenic flies expressing hTPM4. Crizotinib treatment increased the survival of hTPM4-overexpressing flies at 200 nM (HR: 0.556; *P*<0.01) and 1 μM (HR: 0.523; *P*<0.01) (Fig. 3C, Table 1). The mean lifespan of hTPM4-expressing flies treated with 200 nM or 1 µM crizotinib was 33.42±1.02 and 33.42±1.08 days, respectively, compared with 27.36±1.20 days for untreated hTPM4-expressing flies. The maximum lifespan of the hTPM4-expressing flies treated with 200 nM or 1 µM crizotinib was 39 days (Table 2), the same as the untreated hTPM4-expressing flies.

### Crizotinib failed to increase the lifespan of transgenic *Drosophila* expressing hALK

We investigated the effect of crizotinib on the lifespan of hALK transgenic flies expressing full-length wild-type hALK. Despite being an inhibitor of ALK, treatment with 200 nM or 1 µM crizotinib had no effect on the lifespan of these flies (HR: 0.969; *P*=0.877; HR: 0.921; *P*=0.680) (Fig. 3D, Tables 1 and 2).

### Crizotinib inhibits the constitutive activity of hTPM4-hALK in *Drosophila*

**Fig. 4.**
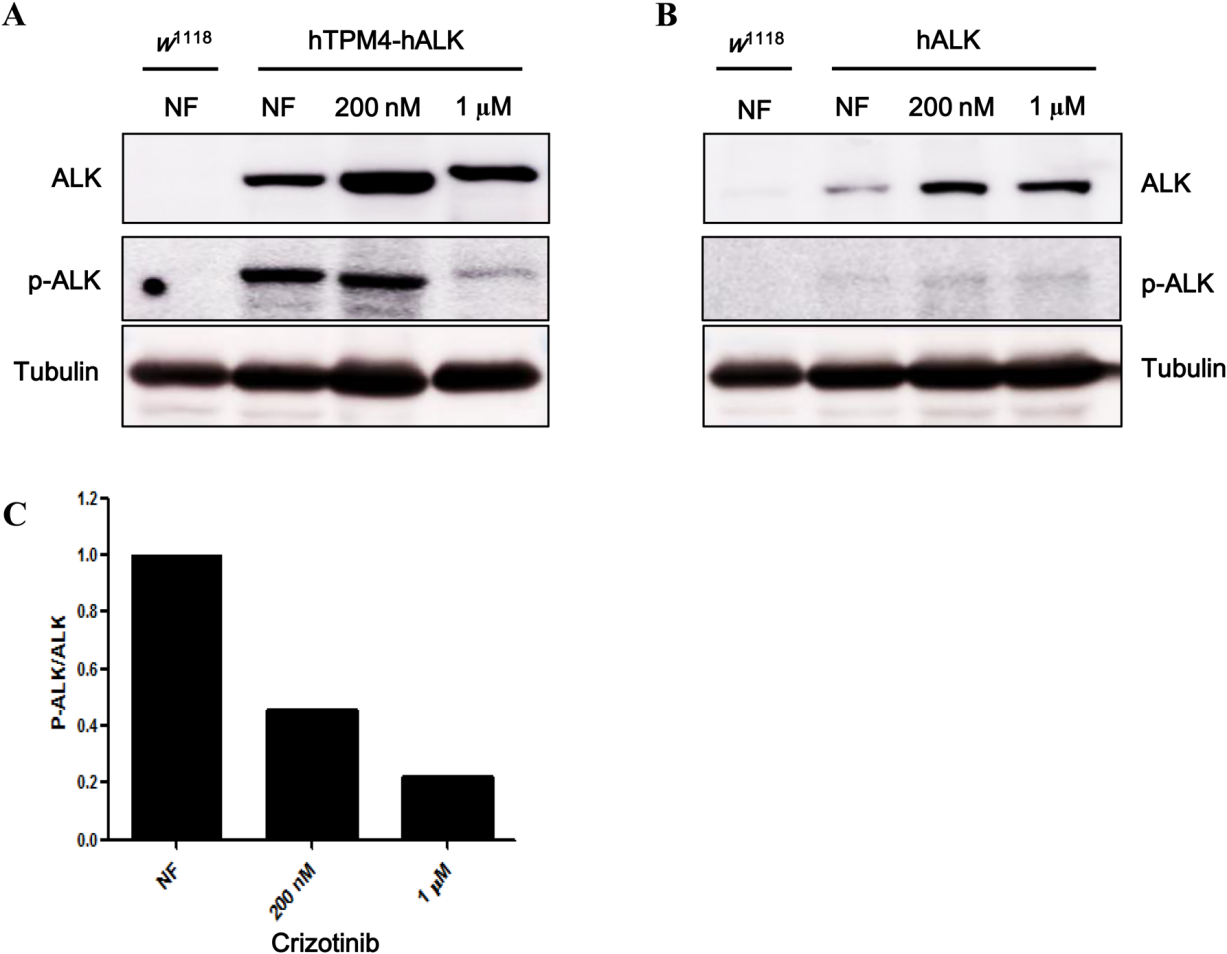
**The effect of crizotinib on ALK phosphorylation of transgenic flies expressing hTPM4-hALK or hALK.** Western blot analysis of the levels of total hALK and hALK phosphorylated at Tyr^1278^ in flies overexpressing hTPM4-hALK or hALK. (A) hTPM4-hALK was phosphorylated at Tyr^1278^ in a ligand-independent manner. The flies expressing hTPM4-hALK showed a decreased level of phosphorylated Tyr^1278^ after crizotinib treatment compared with no treatment, with a greater decrease induced by 1 µM compared with 200 nM crizotinib. (B) Wild-type hALK was slightly phosphorylated. (C) Quantitation of the western blot results in A is represented graphically. NF, normal food.

TPM3-ALK and TPM4-ALK are constitutively active oncogenes acting independently of ligands and tyrosine phosphorylation (Lawrence et al., 2000). Autophosphorylation of Tyr^1278^ is necessary for full activation of the ALK domain, as well as the transformation ability of ALK fusion proteins (Tartari et al., 2008). Therefore, we measured the level of phosphorylation at Tyr^1278^ within the hTPM4-hALK fusion protein in transgenic *Drosophila* treated with crizotinib*.* As shown in Fig. 4A, hTPM4-hALK was phosphorylated at Tyr^1278^ in a ligand-independent manner, and flies treated with crizotinib exhibited a decreased level of phosphorylated Tyr^1278^ within hTPM4-hALK compared with untreated flies, and this effect was more pronounced with 1 µM compared with 200 nM crizotinib (Fig. 4A,C). These results were consistent with the prolonged survival of hTPM4-hALK-expressing flies treated with crizotinib. Taken together, the results suggest that crizotinib increased the lifespan of *Drosophila* by inhibiting the constitutive activity of hTPM4-hALK.

## DISCUSSION

Numerous genes and pathways associated with cancer have been identified, and molecular-targeted therapies have been developed using various model systems (Yadav et al., 2016; Geng et al., 2017). We analyzed the effects of crizotinib using *Drosophila* because of the numerous advantages of this model organism (Sun et al., 2013; Yadav et al., 2016). Crizotinib was originally developed as a selective c-MET inhibitor by Pfizer. However, after testing on a large panel of 120 kinases, it was found that crizotinib also potently inhibits ALK and induces apoptosis in anaplastic large-cell lymphoma (Christensen et al., 2007; Zou et al., 2007).

Previously, we identified the TPM4-ALK fusion protein in patients with gastric cancer (unpublished). To characterize the influence of ALK overexpression on the effect of crizotinib, we generated transgenic *Drosophila* overexpressing hALK, hTPM4, or hTPM4-hALK and analyzed the lifespan of these flies following crizotinib treatment.

Crizotinib dose-dependently extended the lifespan of hTPM4-hALK-overexpressing flies. However, there was little effect on hALK-expressing flies, despite crizotinib being an inhibitor of ALK. These results were similar to those of Fang et al. (2014), who investigated the effect of crizotinib in a patient-derived xenograft model. Tumors harboring the HIP1-ALK fusion gene sensitively responded to crizotinib, whereas tumors harboring wild-type ALK did not. Because crizotinib inhibits ALK phosphorylation and cell proliferation (Ou, 2011), we investigated the level of phosphorylated (Tyr^1278^) ALK using western blot analysis. Wild-type hALK was only slightly phosphorylated because of insufficient ligand stimulation, whereas hTPM4-hALK was phosphorylated in a ligand-independent manner (Fig. 4A,B). Furthermore, the increased survival of hTPM4-hALK-expressing flies was correlated with the decreased level of Tyr^1278^ phosphorylation (Figs 3B and 4A). Taken together, our data suggest that wild-type hALK is resistant to crizotinib treatment.

We also generated hTPM4-expressing transgenic *Drosophila* as drug treatment controls. The survival of hTPM4-expressing flies was decreased compared with *w*^1118^ flies. Unexpectedly, although crizotinib is an inhibitor of ALK, the survival of hTPM4-expressing flies was rescued by crizotinib. The crizotinib-induced increase in survival was similar to that in *w*^1118^ flies and greater than that in untreated hTPM4-expressing flies (Fig. 3C, Table 1). Taken together, it is possible that crizotinib acts via hTPM4. Both decreased and increased TPM4 expression has been observed in human cancers, depending on the tumor type (Stehn et al., 2006). Inhibitors of cancer-associated TPMs, and actin-targeting compounds, have been developed to disrupt the actin cytoskeleton and actin polymerization in tumor cells (Bonello et al., 2009; Stehn et al., 2013). However, anti-actin drugs induce cardiac and respiratory muscle toxicities (Schweikart et al., 2013). Thus, novel TPM inhibitors that specifically target actin have been developed (Bonello et al., 2016; Currier et al., 2017). Further studies will be required to determine the mechanism by which crizotinib affects TPM4, to support the value of crizotinib as a therapeutic agent without cardiac and respiratory muscle toxicities.

In summary, this study is the first to assess the effects of crizotinib on the lifespan of *Drosophila* overexpressing hTPM4-hALK, hALK or hTPM4. Our results showed that crizotinib may be an effective treatment for cancers overexpressing hTPM4-hALK, and hTPM4 may be a crizotinib target gene.

## MATERIALS AND METHODS

### Genetics and *Drosophila* growth conditions

The hTPM4, hALK and hTPM4-hALK genes were cloned into the pUAST germline transformation vectors (Rubin and Spradling, 1982), which were then co-injected with helper plasmids encoding the p-element into *Drosophila* embryos to create transgenic *Drosophila*. The transformants were selected and further characterized. The GAL4/UAS system was used to overexpress the target gene in tissues and cells, and Tubuline-GAL4 driver flies were used to express target genes. The flies were cultured on standard agar media at 25±1°C under 60±1% relative humidity.

### Fly food formulation

All experimental files were reared on a standard *Drosophila* medium consisting of 1 l dH_2_O, 7.7 g agar, 62.4 g dried yeast, 40.8 g corn starch, 84.0 g glucose, 13.0 ml molasses and 12.5 ml mold inhibitor (DaeJung Chemicals & Metals, Seoul, Republic of Korea). Crizotinib (PF-02341066) was added to the feed at final concentrations of 200 nM or 1 μM.

### Western blot analysis

Cell lysates were obtained from approximately ten male or female flies by grinding in 300 μl RIPA buffer supplemented with proteinase and phosphatase inhibitors. The lysates were centrifuged at 13,000 rpm for 20 min at 4°C, and protein concentrations were determined using the BCA method.

Samples containing 30–50 μg total protein were resolved on a 10% SDS-PAGE gel and transferred to a nitrocellulose membrane. The membrane was blocked with 5% skim milk in 1× TBS-T and incubated with the following primary antibodies: anti-TPM4 (1:100; Developmental Studies Hybridoma Bank, Iowa City, IA, USA), anti-ALK (1:250; Santa Cruz Biotechnology, Santa Cruz, CA, USA), anti-pALK (1:1000; Cell Signaling Technology, Danvers, MA, USA), and anti-α-tubulin (1:5000; Developmental Studies Hybridoma Bank). The membrane was incubated at room temperature with secondary antibodies at the appropriate dilutions. Quantitation of the western blot results is represented graphically using ImageJ software (National Institutes of Health, Bethesda, MD, USA).

### qRT-PCR analysis

Total RNA was extracted from five male or female flies using TRIzol reagent (Ambion, Waltham, MA, USA), and 1 μg total RNA was used for cDNA synthesis using the PrimeScript™ RT Reagent Kit (Takara Bio, Kusatsu, Japan). Each reaction mixture contained 5 μl SYBR Green PCR Master Mix (Takara Bio), 0.4 μl forward and reverse primers (10 pmol each), cDNA (1:6 dilution), and diethyl pyrocarbonate-water to reach a final volume of 10 μl. The mRNA levels of the target genes were normalized to that of the *Drosophila rp49* gene and were analyzed using the 2^-ΔΔCT^ method with Excel software (Microsoft, Redmond, WA, USA). The reactions were performed in duplicate. The following primers were used for PCR: 5′-TTG AGG AGG AGT TGG ACA GG-3′ and 5′- GCT GCA TCT CCT GAA TCT CC-3′ for hTPM4, 5′-GCA ACA TCA GCC TGA AGA CA-3′ and 5′- GCC TGT TGA GAG ACC AGG AG-3′ for hALK, and 5′-ACG GTT GCA AAA CTG GAA AA-3′ and 5′-TTG GGG TTG TAG TCG GTC AT-3′ for hTPM4-hALK.

### Drug treatments and lifespan measurements

The method of measuring lifespan to determine the effects of genetic and non-genetic factors was described previously (Sun et al., 2013). A total of 100 flies (50 male and 50 female) were collected at 3 days after eclosion (ten flies per vial). The flies were kept at 31±1°C for survival analysis. The surviving flies were transferred every 3 days to new vials containing the appropriate normal food or food spiked with crizotinib (200 nM or 1 µM).

This practice was continued until all flies were dead. HRs and *P*-values were calculated using Kaplan–Meier survival analysis (Dinse and Lagakos, 1982). The HRs with 95% confidence intervals were compared using the log-rank test, and *P*-values were calculated (Mantel, 1966). The survival curves and statistical analyses were performed using the R program package (www.rproject.org) as described previously (Kim et al., 2015). The maximum lifespan was calculated as the average age of the top 10% longest-living flies (Lushchak et al., 2012).
